# Primary pleural hydatidosis presenting as an isolated cough: A rare case report

**DOI:** 10.1016/j.ijscr.2023.108533

**Published:** 2023-07-26

**Authors:** Marlyn Susan George, Preethi Jagannath, Tigran Byuzandyan, Natalie Baghumyan, Armen Khanoyan, Md Foorquan Hashmi

**Affiliations:** a Yerevan State Medical University, Armenia, Mkhitar Heratsi 18A, Yerevan 0095, Armenia; b M S Ramaiah Medical College, Bangalore, India; c National Center of Oncology, Yerevan, Armenia; d Yerevan State Medical University, Armenia, Yerevan, Armenia

**Keywords:** Case report, Primary pleural hydatidosis, Isolated cough, Thoracotomy, Pleural effusion

## Abstract

**Introduction and importance:**

Echinococcus granulosus causes hydatid disease. The most affected organ is the liver which is followed by the lungs. The pleural cavity being the primary location of hydatid cysts is rare and should be discussed further. This paper documents a rare case of primary pleural hydatidosis which can present with a merely isolated cough followed by dyspnea. The diagnosis and surgical treatment along with post-operative medications are vital in this case.

**Case presentation:**

We present a case of a 45-year-old who suffered from a cough for more than one week which did not subside after taking medications. This symptom was followed by dyspnoea for which an X-ray was done which showed left-sided pleural effusion, a complication of pleural hydatidosis. Computed tomography showed multiple cysts in the pleural cavity which confirmed the diagnosis of primary pleural hydatidosis as the cysts were not present in any other sites. Blood work revealed eosinophilia which is significant in parasitic diseases. A left posterolateral thoracotomy was performed, and the cysts were surgically removed. Additionally, empyemectomy and pleurectomy were done. The patient was then treated with anti-parasitic therapy and was advised to get X-rays during the follow-up visits. The X-rays were normal and indicated that there was no disease recurrence.

**Clinical discussion:**

Echinococcus granulosus is a parasitic worm that causes hydatid disease. The primary location is the liver. A diagnosis of intrathoracic but extrapulmonary disease, which involves the presence of hydatid cysts in the pleura, heart, pericardium, mediastinum, chest wall, and diaphragm, is difficult in individuals lacking a primary cyst in a common location (Isitmangil et al., 2003; Saeedan et al., 2020).

**Conclusion:**

This case implies the significance of a cough of more than a week that is not relieved by medications. This should be carefully evaluated and followed in cases that have a rare diagnosis requiring surgery. A diagnosis of primary pleural hydatidosis with left-sided pleural effusion and atelectasis with mediastinal shift to the right side was made which was treated with a surgical procedure.

## Highlights

• Primary pleural hydatidosis can present with merely an isolated cough.
• Pleural effusion is one of the complications of ruptured pleural hydatid cysts.
• Posterolateral thoracotomy is done to remove the hydatid cysts.
• Pleurectomy and empyemectomy are done in infection-induced pleural effusion.

## 1 Introduction

Although hydatid cysts were noticed in both livestock and people in ancient times, it wasn't until the 17th century that their biological origin became clear. The parasites that form hydatid cysts are members of the genus Echinococcus. They are closely related cestodes that have adapted to a diverse range of host populations connected by predator and prey interactions [10].

Hydatid disease caused by Echinococcus granulosus typically requires two hosts: one intermediate host (humans, cows, sheep) and one definitive host (wolves, foxes, dogs) [2,3,8].

About 60–80 % of hydatid cysts are found in the liver. The second most common location is the lung which has been seen in 10–30 % of cases of hydatidosis. A hydatid cyst in the pleural cavity as the primary location is very rare [4].

One of the complications of a ruptured pleural hydatid cyst is a massive pleural effusion associated with atelectasis and mediastinal shift to the opposite side of the chest [3].

We present a rare case of a patient who came with a mere presentation of an isolated cough for more than 1 week. This cough was not relieved by any medications. It was followed by a short presentation of dyspnoea for which an X-ray and a Computed tomography were done and a diagnosis of primary pleural hydatidosis was made with a complication of pleural effusion. He was taken to the operation room for surgical removal of the hydatid cysts from the pleural cavity.

## 2 Case presentation

A 45-year-old male presented to the emergency department complaining of a cough for more than a week. This cough was not relieved by any medications. It was followed by dyspnoea for 2 days. From the patient's history, we found that he is a schoolteacher from a middle-class family. The living conditions of the family are at subpar levels. He is a non-smoker and an occasional alcohol consumer. There is no evidence of contact with domestic animals like dogs, sheep, or cows. The patient lives with his wife and two children in his home with no domestic animals as pets or livestock. Contamination of food and water are a few environmental risk factors that the patient has.

On physical examination, the patient had a normal heart rate, blood pressure, oxygen saturation, and temperature. His respiratory rate was significant for tachypnoea with 25 cycles per minute. On clinical examination, breath sounds were decreased on the left side, also vocal fremitus was found to be decreased, along with dullness on percussion on the left side of the chest. The right side of the chest showed normal clinical examination. Laboratory findings were significant for eosinophilia (12 %). A routine chest X-ray revealed rounded opacities and a large left-sided pleural effusion with a right-sided mediastinal shift. It did not reveal consolidation of the upper zone with ipsilateral hilar enlargement or any features suggestive of pulmonary tuberculosis.

The patient was hospitalized urgently and was referred to the Respiratory Medicine Department for further evaluation and management. The patient was scheduled for a pleural drainage procedure. A pleural drainage was inserted in the 5th intercostal space anterior to the mid-axillary line on the left side of the chest. 2600 ml of cloudy yellowish fluid was drained within 24 h. A differential diagnosis of tuberculosis, malignancy, and parapneumonia was considered based on the imaging which showed large left-sided pleural effusion and exudative pleural effusion on seeing the cloudy yellowish fluid on drainage. The fluid drained was sent for protein, lactate dehydrogenase, Gram stain, cytology, and microbiological culture. The pleural fluid analysis confirmed an exudative pleural effusion.

Computed tomography was done to confirm the suspicion of hydatid disease. The CT revealed multiple small cysts which are round in shape filling up the entire left pleural cavity. Left-sided pleural effusion was associated with left lung atelectasis and right mediastinal shift. Abdominal CT was done which showed no lesion in the liver suggestive of the absence of a primary foci of hydatidosis in the liver. The patient was referred to the Surgery Department for further management of the case. Preoperative antihelminthic treatment with Albendazole 15 mg/kg/day for 6 days was completed**.** He completed the pre-anesthetic check-ups and was posted for surgery by the Chief Surgeon.

**Fig. 1–3:**
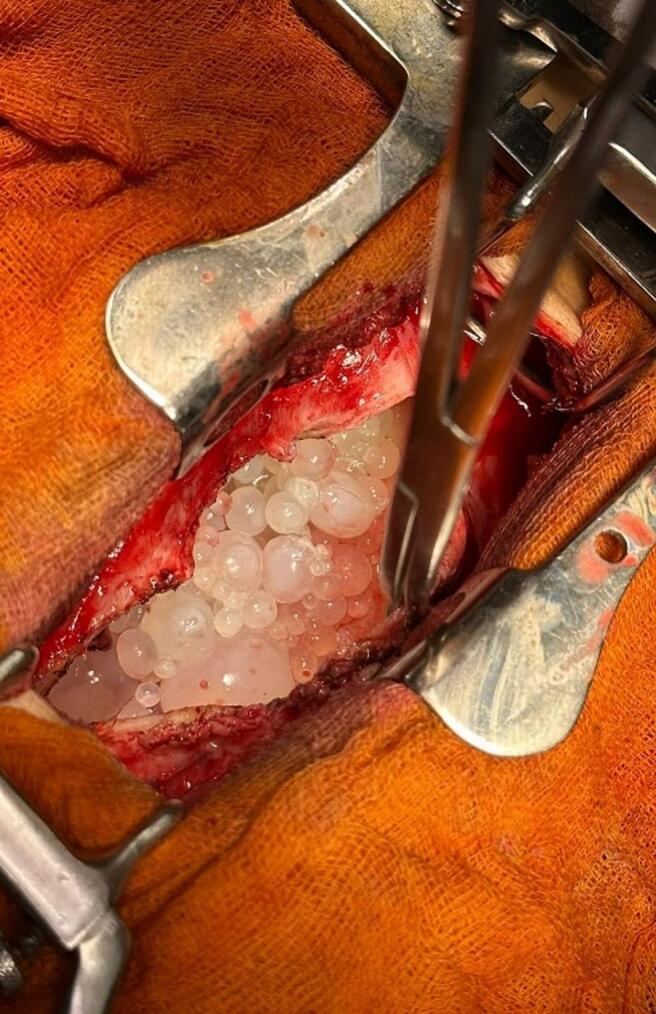

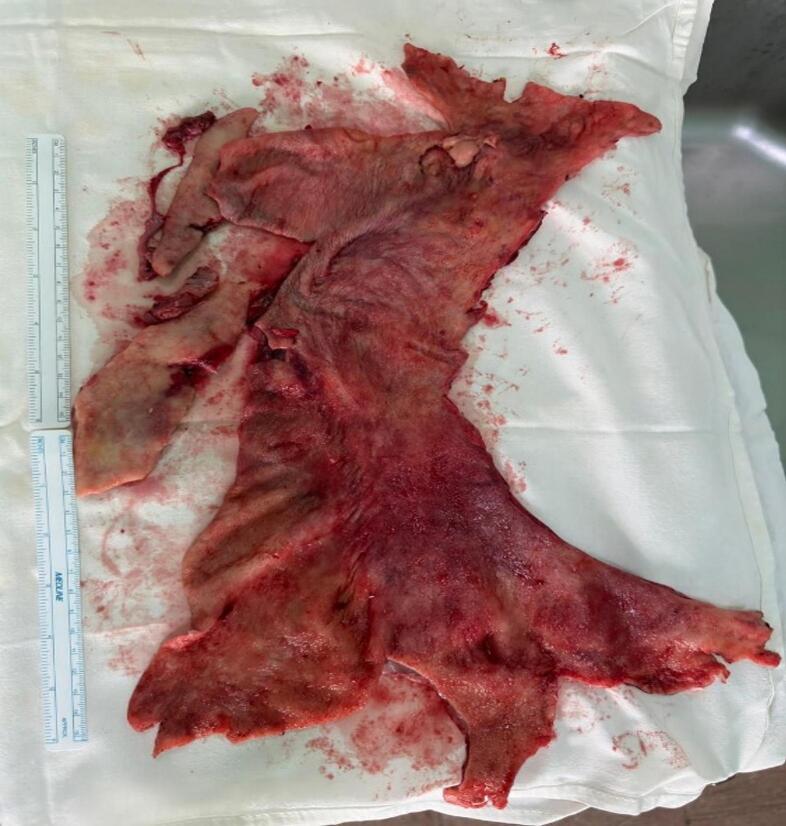

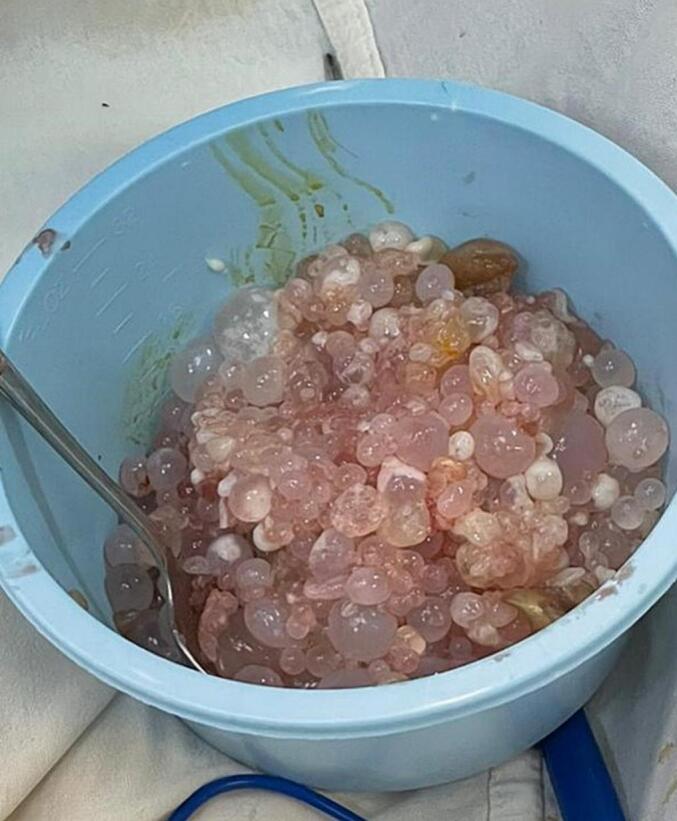
Images of multiple daughter cysts and pleural after pleurectomy.

The patient was taken to the operation room for a left-sided posterolateral thoracotomy in the 5th intercostal space. The pleural space was then irrigated with hypertonic saline to kill the scolices. Multiple daughter cysts were removed, and a thorough inspection showed no other cysts in the pleural space, lung, diaphragm, or mediastinum (Fig. 1–3).

A pleurectomy and an empymectomy were done in view of infection-induced pleural effusion. After the procedure was done, the collapsed lung was expanded fully, and no air leakage was found. Additionally, there was no sign of parenchyma damage to the lung. It was followed by the closure of the thoracotomy. The patient was shifted to the Intensive Care Unit and was then discharged when he was hemodynamically stable and symptomatically better with 10 mg/kg/day of oral Albendazole for 1 year. The patient was asymptomatic and showed normal chest X-rays in the follow-up visits done monthly and yearly.

## 3 Discussion

Echinococcus granulosus is a parasitic worm that in adult form resides in the jejunum of dogs and other canines and lays eggs that are expelled in the stool [1].

The intermediate hosts (humans, cows, sheep) ingest the eggs, which then release embryos that enter the portal circulation. These embryos mostly reside in the liver but also tend to spread to different organs in the body. A diagnosis of intrathoracic but extrapulmonary disease, which involves the presence of hydatid cysts in the pleura, heart, pericardium, mediastinum, chest wall, and diaphragm, is difficult in individuals lacking a primary cyst in a common location [2,5].

Primary pleural hydatidosis is the presence of hydatid cysts primarily in the pleural cavity. Pleural effusion, pneumothorax, hydropneumothorax, empyema, pleural thickening, and collapsed lung can result from cysts at this location rupturing and releasing their contents into the pleural cavity. One of the complications which is rare is an anaphylactic shock due to the rupture of a pleural or a lung hydatid cyst [[5], [6], [7]].

In our case, the cysts were found only in the pleural cavity for which left-sided posterolateral thoracotomy in the 5th intercostal space was done to kill the scolices and remove the cysts. Additionally, we drained the parasitic pleural effusion. Because we were unable to locate any extra pleural lesions, we concluded that the diagnosis was primary pleural hydatidosis, which is an incredibly uncommon clinical condition. Pleural hydatidosis can resemble the clinical symptoms of pleural effusions such as dyspnoea, chest discomfort, dry cough, and a mediastinal shift to the opposite side. However, in 15 % of the cases, it can be asymptomatic [8].

In our case, the patient presented with a cough for more than 1 week which did not subside with medications. It was followed by shortness of breath. When evaluated, the X-ray showed a left-sided pleural effusion and a lung collapse on the same side of the effusion. Computed tomography showed multiple cysts in the entire left pleural area, causing a mediastinal shift to the right.

Radiological imaging is an important modality for the diagnosis of pleural hydatidosis. Most often, a chest X-ray and a CT scan can assist in confirming the diagnosis and plan for surgery, which is the primary treatment for hydatid disease in addition to postoperative antiparasitic therapy [10].

Skin tests like Casoni's test, indirect hemagglutination test, and complement fixation tests can be used for diagnostic purposes. We rarely use them routinely because of their false-positive results.

When the pulmonary or primary pleural hydatidosis is bilateral, a median sternotomy with resection of cysts is the choice of surgery. Whereas a thoracotomy and resection of cysts on the same side of the disease are preferred in unilateral cases [11].

Staged Thoracotomy was not preferred in our case as it is usually used in bilateral pleural hydatidosis cases. Video-assisted thoracoscopic surgery, a minimally invasive procedure was not used here as it was not feasible to remove such multiple cysts from the thoracic cavity and as the risk of rupture of the cysts was high.

In our case, the patient was successfully treated with a left posterolateral thoracotomy followed by irrigation with a hypertonic solution. In addition to resection of all the cysts from the pleural cavity, pleural drainage, empyemectomy, and pleurectomy were done.

A table comparing this case with the previously reported cases of primary pleural hydatidosis is added below to highlight the novelty of our case:

**Table:** 

Other case reports on Primary Pleural Hydatidosis	The novelty of our case on Primary Pleural Hydatidosis
Occupational history is significant. The patient works as a shepherd.	Our patient works as a school teacher. There is minimal to no exposure to Echinococcus organisms.
The patient presents with a wide variety of symptoms like chest pain, dyspnea, weight loss	The patient presented with an isolated cough when he came to the Emergency room.

## 4 Conclusion

Primary pleural hydatidosis which is a very rare disease can have a mere presentation of cough followed by dyspnoea. In our case, the X-ray showed a left-sided pleural effusion, a complication of ruptured pleural hydatid cysts. Hydatid disease was confirmed on a CT scan. A left posterolateral thoracotomy was performed on the patient. A full recovery of the lung's size and functionality offered the prognosis of a very positive outcome. To identify recurrence, it is crucial to do a CXR on follow-up visits. It is also vital to provide anti-parasitic drugs.

## 5 Methods

Work has been reported in line with CARE criteria.

## Consent for publication

Written informed consent was obtained from the patient for the publication of this case report and accompanying images.

## Ethical approval

Not applicable.

## Funding

Not applicable.

## Author contribution

Marlyn and Dr.Preethi wrote the Manuscript,Dr.Tigran and Dr.Armen provided and cared for study patients. Foorquan and Dr.Natalie made the final draft

## Guarantor

Marlyn Susan George.

## Research registration number

N/A.

## Conflict of interest statement

The authors declare that they have no competing interests.

## Data Availability

The datasets are available from the corresponding author upon reasonable request.

## Appendix A Supplementary data

Media 1 & 2 CT showing multiple small cysts filling up the entire left pleural cavity. Media 1 & 2

Media 3 A video showing multiple daughter cysts being removed. Media 3

Media 4 Timeline Image 1
