# Supplementary material for: Primary pleural hydatidosis presenting as an isolated cough: A rare case report
Source: Int J Surg Case Rep. 2023 Jul 26;109:108533. doi: 10.1016/j.ijscr.2023.108533 (PMC10400858; doi:10.1016/j.ijscr.2023.108533)
Supplement: Media 4 — Timeline [file mmc4.docx]

Day 1

Day 1

Day 3

Day 4

Day 5

**Diagnostic Evaluations**

- Laboratory findings: Eosinophilia (12%)
- Routine Chest X-ray: a left-sided pleural effusion with numerous round-shaped opacities in it.
- A left-sided posterolateral thoracotomy was done in the 5th intercostal space.
- Pleural space was irrigated with hypertonic saline to kill the scolices.
- Multiple daughter cysts were removed.
- A pleurectomy and an empymectomy were done in view of infection-induced pleural effusion.
- The collapsed lung was expanded fully, and no air leakage was found.
- Patient was shifted to ICU for observation.
- Patient was discharged on 10mg/kg/day of oral Albendazole for 1 year.

**Physical and Clinical Examination**

- Vitals: normal heart rate, blood pressure, oxygen saturation, temperature. Respiratory rate – 25 cycles per minute.
- On palpation of the chest: Vocal fremitus was decreased on the left side.
- On percussion of the chest: dullness was found on the left side.
- On auscultation of the chest: Breath sounds were decreased on the left side.

**Current illness**

- > 1 week of cough not relieved with medications.
- Dyspnoea for 2 days

**Relevant history**

45-year-old male, a non-smoker, and an occasional alcohol consumer

Asymptomatic patient with normal chest X-rays in follow-up visits

- Computed tomography confirmed hydatid disease which revealed multiple small cysts filling the entire left pleural cavity. Left-sided pleural effusion was associated with left lung atelectasis and right mediastinal shift.
- Pleural drainage procedure was done draining 2.6L cloudy yellowish fluid within 24 hours.
